# Advances in Skin Substitutes—Potential of Tissue Engineered Skin for Facilitating Anti-Fibrotic Healing

**DOI:** 10.3390/jfb6030547

**Published:** 2015-07-09

**Authors:** Mathew Varkey, Jie Ding, Edward E. Tredget

**Affiliations:** 1 Wound Healing Research Group, Division of Plastic and Reconstructive Surgery, University of Alberta, 2D3.81 WMSHC, 8440-112 Street, Edmonton, AB T6G 2B7, Canada; E-Mails: mvarkey@ualberta.ca (M.V.); jied@ualberta.ca (J.D.); 2 Critical Care Medicine, University of Alberta, 2D3.81 WMSHC, 8440-112 Street, Edmonton, AB T6G 2B7, Canada

**Keywords:** skin substitutes, tissue engineering, wound healing, fibrosis

## Abstract

Skin protects the body from exogenous substances and functions as a barrier to fluid loss and trauma. The skin comprises of epidermal, dermal and hypodermal layers, which mainly contain keratinocytes, fibroblasts and adipocytes, respectively, typically embedded on extracellular matrix made up of glycosaminoglycans and fibrous proteins. When the integrity of skin is compromised due to injury as in burns the coverage of skin has to be restored to facilitate repair and regeneration. Skin substitutes are preferred for wound coverage when the loss of skin is extensive especially in the case of second or third degree burns. Different kinds of skin substitutes with different features are commercially available; they can be classified into acellular skin substitutes, those with cultured epidermal cells and no dermal components, those with only dermal components, and tissue engineered substitutes that contain both epidermal and dermal components. Typically, adult wounds heal by fibrosis. Most organs are affected by fibrosis, with chronic fibrotic diseases estimated to be a leading cause of morbidity and mortality. In the skin, fibroproliferative disorders such as hypertrophic scars and keloid formation cause cosmetic and functional problems. Dermal fibroblasts are understood to be heterogeneous; this may have implications on post-burn wound healing since studies have shown that superficial and deep dermal fibroblasts are anti-fibrotic and pro-fibrotic, respectively. Selective use of superficial dermal fibroblasts rather than the conventional heterogeneous dermal fibroblasts may prove beneficial for post-burn wound healing.

## 1. Introduction

**Figure 1:**
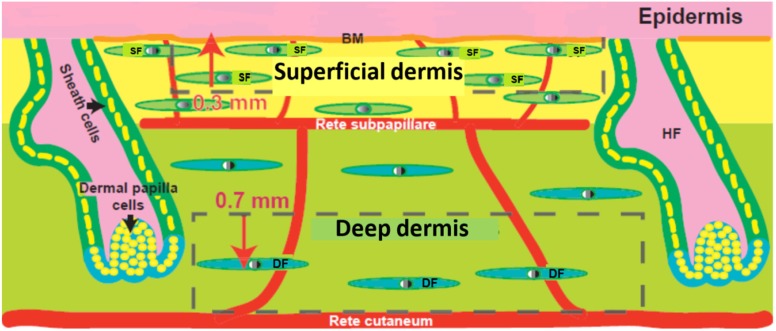
Structure of human skin showing dermal heterogeneity. Epidermis, basement membrane (BM) superficial dermis, rete pappillare, deep dermis and rete cutaneum. SF: superficial fibroblasts, DF: deep fibroblasts, HF: hair follicle are indicated. Modified from [15].

Skin, which is the largest organ of the body, plays a primary role in protecting the body from mechanical damage such as wounding. It comprises of epidermal, dermal and hypodermal layers (Figure 1). The barrier function of the skin is provided by its avascular epidermal layer, which is composed mainly of keratinocytes. The keratinocytes form a stratified epithelium, with proliferating basal cells at the innermost layer and the keratinized, relatively impermeable outer stratum corneum layer on the surface [1]. Other non-epithelial epidermal cells include melanocytes that provide skin pigmentation; Langerhans cells that are antigen-presenting dendritic cells of immune system; and Merkel cells that are thought to function as mechanoreceptors. The dermal layer, which is the layer below the epidermis, is highly vascular, provides structural integrity and forms the bulk of the skin [1]. It is composed of type I collagen with some elastin and glycosaminoglycans (GAGs), which cushion the body against mechanical injury by conferring elasticity and plasticity to the skin. Fibroblasts, the main dermal cell type, produce remodelling enzymes such as proteases and collagenases that play important roles in the wound healing process [2]. There are two distinct layers of fibroblasts, the papillary or superficial fibroblasts that lie next to the basal epidermal layer and the reticular or deep fibroblasts that lie deeper inside. Depending on age and anatomical location the superficial dermis is generally approximately 300–400 µm deep and extends till the rete subpapillare, the vascular plexus that marks the lower limit of the papillary dermis. On the other hand, the reticular dermis extends from the rete subpapillare to the rete cutaneum, a deeper vascular plexus that demarcates the dermis and the hypodermis. The other cells in the dermis include endothelial cells, smooth muscle cells and mast cells. The dermal cells are embedded on the extracellular matrix (ECM), which serves as a scaffold to bind, integrate and support cells. ECM is a complex mixture of structural and functional proteins arranged in a unique three-dimensional ultrastructure that fills the extracellular space between cells [3,4]. ECM regulates cellular growth via two major classes of macromolecules: the GAGs, which are predominantly linked to proteins to form proteoglycans, and the fibrous proteins [5,6]. The proteoglycans can be either secreted into the extracellular environment as in the case of chondroitin sulphate and hyaluronic acid, or anchored on the plasma membrane as syndecan-1 [4]. The fibrous proteins can be structural (collagen type I and elastin), adhesive (fibronectin) or de-adhesive (tenascin-C and thrombospondin) in nature [7]. The ECM of basement membrane present immediately beneath epithelial cells comprises of distinctly different collections of collagenous and non-collagenous proteins including laminin, collagen type IV and entactin [8,9]. The basement membrane contains laminin, nidogen, collagen types IV and VII, and the proteoglycans, perlecan and collagen XVIII, in addition to the extracellular matrix proteins: collagen type I and III, tenascin, and fibrillin-1 [10,11]. In the basement membrane, collagen type IV, laminin-5, nidogen, and perlecan form a network that functions as a barrier between the epidermis and dermis. Collagen type VII, another essential component of the basement membrane, forms fibrils that anchor the basement membrane to the underlying dermis. Both keratinocytes and fibroblasts contribute to the formation of the basement membrane [12,13,14]. The basement membrane and the underlying dermis play critical roles in the maturation and function of skin by regulating keratinocyte growth and terminal differentiation. The barrier property of the epidermis is due to the presence of tight junctions and lipids, such as ceramides, cholesterol esters, and free fatty acids, which accumulate during epidermal differentiation. The third layer of skin, the hypodermis, contains adipose tissue that is well vascularized and contributes to both thermoregulatory and mechanical properties [1]. The hypodermis is also known be reservoir of regulatory factors known as adipokines; some of which have anti-inflammatory properties and have effects on the dermis; the hypodermal hormone adiponectin enhances synthesis of hyaluronic acid by dermal fibroblasts. The integrity of the three layers of skin is critical for maintenance and the proper functioning of the skin as well as survival. 

## 2. Skin Injury from Burns

Human skin performs a wide range of functions including perception, regulation of water and temperature loss, and importantly provides a protective barrier that is most critical for our survival. When the skin is compromised during injury as in the case of acute burn wounds or chronic wounds such as pressure and leg ulcers, the skin needs immediate coverage so as to facilitate regeneration and repair. Burn wounds are caused by damage to the skin due to heat, chemicals, electricity or radiation. Burns that result in damage to the epidermal, dermal (papillary and reticular) and hypodermal layers of the skin are referred to as first, second and third degree burns, respectively. Second and third degree burns cause fluid loss, drastic disturbances of ionic equilibrium, loss of temperature control, pain, immunodepression, bacterial invasion and in some cases substantial or permanent disability. According to the latest statistics, annually in the United States 450,000 burn injuries require medical attention with 40,000 of these patients requiring hospitalization. The cost burden on the healthcare system due to severe burn injuries is significant, with per patient hospital charges alone ranging from $27,000 for burns affecting less than 30% total body surface area to more than $500,000 for more severe burns [16].

The most common treatment for patients with burns and other skin wounds is the use of skin grafts. In the case of severe burn injuries, permanent wound closure currently requires grafting of autologous epithelium, wherein an area of suitable skin is separated from the tissue bed and transplanted to the recipient area on the same individual from which it should receive new blood supply. The autografts may be full-thickness, wherein a complete section of the epidermis and dermis is transplanted, or split-thickness where only part of the dermis is used [17]. Use of split-thickness skin autografts is the gold standard for restoration of epidermal function of the skin in burn patients [18]. However, the difficulty in the treatment of patients with extensive and deep burns is the limited availability of sufficient donor sites for autografting. In addition, autografting generates donor sites which are not only painful during healing, but may also scar and become a cause of long-term morbidity [19]. The other grafting procedures besides autografting include syngeneic, allogeneic and xenogeneic skin grafts. Syngeneic grafting is performed between genetically identical individuals such as monozygotic twins, and is taken equally well as autografts. Allogeneic grafting involves skin transplantation from non-genetically identical individuals of the same species or cadaver skin, whereas xenogeneic grafting involves transfer of skin between species. These grafts serve only as temporary treatments for full thickness burns since they require resurfacing with an autogenous epidermal layer because of immunologic rejection. Also, as opposed to autografts, both allografts and xenografts are often rejected since the antigens present in the donor tissue elicit host immune response [20]. Overall, there are several limitations associated with the use of different types of grafts for treatment of patients with extensive skin loss; hence, there is an immense need to develop alternative therapeutic options.

## 3. Skin Substitutes

Skin substitutes are artificial skin replacement products that provide the protective barrier of the skin when placed over acute burn injuries or other chronic skin wounds such as cutaneous ulcers and congenital anomalies such as giant nevus [17,21,22,23,24]. Their primary objective is to work as skin equivalents, facilitate repair and regeneration, and restore the functional properties of skin. Several skin substitutes have been useful for replacement or reconstruction of one or both layers of the skin, facilitating wound healing in several different clinical settings. These skin substitutes can act as temporary wound covers or permanent skin replacements, depending on their design and composition. They reduce or remove inhibitory factors, and help in providing rapid and safe coverage. The main advantage of using skin substitutes is that they reduce or eliminate the need for donor site area, which is required for autologous split-thickness grafts. This makes skin replacement procedures available to patients contraindicated for autologous grafts such as those with over 60% of total body surface area (TBSA) burned, smoke inhalation injured, and the very young and elderly. Skin substitutes also decrease the patients’ risk of infection and sepsis especially at the thin dermis of the donor site [17,21,22,23,24]. They pose negligible risk of cross-infection, which is not the case with allografts and xenografts. Furthermore, skin substitutes reduce mortality and morbidity from scarring (both at donor and treatment sites), changes in pigmentation and patients’ burden of pain. More importantly, they reduce the total number of surgical procedures required and patient hospitalization time [22,23].

## 4. Features of Skin Substitutes 

Some of the essential features of a skin substitute are that it should be sterile, provide barrier function, allow water vapor transmission similar to normal skin, evoke minimal inflammatory response in the patient, and also have no local or systemic toxicity [24]. Some of the other features of a good skin substitute are that it should adhere to the wound surface in a rapid and sustained manner, have appropriate physical and mechanical properties, and undergo controlled degradation [24]. It should be relatively inexpensive, easy to handle and apply onto wound sites, and also be flexible and pliable so that it could conform to irregular wound surfaces. Additionally, it should be impermeable to exogenous bacteria, resistant to linear and shear stresses, and have minimal storage requirements and an indefinite shelf life. Importantly, it should incorporate into the patient with minimal scarring and also facilitate angiogenesis [1].

## 5. Types of Skin Substitutes

Skin substitutes differ in complexity and can be broadly classified into two types: synthetic, which are made up of acellular materials, and natural, which are made up of cellular materials. The synthetic skin substitutes are most basic and designed to function primarily as barriers to fluid loss and microbial contamination. The natural skin substitutes also known as tissue engineered skin are more advanced in nature and are cultured allogeneic or autologous cell suspensions or sheets used alone or along with a dermal matrix. Tissue engineered skin can be classified into three types: those that consist of cultured epidermal cells with no dermal components, those with only dermal components, and those with a bilayer containing both dermal and epidermal components. Although each of these have their own advantages and have applications in burn treatment, none of them can fully simulate native skin.

## 6. Commercially Available Skin Substitutes 

Some of the commercially available skin substitutes that are often used in the treatment of burn injuries and chronic wounds are discussed below in detail (also see Table 1). Among the synthetic acellular skin substitutes, Biobrane™, Integra™, Alloderm™ and TransCyte™ are most commonly used. Examples of frequently used natural skin substitutes with allogeneic cells include Dermagraft™, Apligraf™ and OrCel™, while those with autologous cells include Epicel™.

Biobrane™ is composed of an outer ultrathin silicone film (epidermal analog) and an inner three-dimensional irregular nylon filament (dermal analog) upon which type I collagen peptides are bonded [23]. The semi-permeable silicone surface controls water vapour loss from the wound. Biobrane™ is used as a temporary wound dressing and is removed upon wound healing or when autograft skin is available. It has been shown to be as effective as frozen human allografts and contributes to better healing, when used on excised full-thickness burns [23]. In addition, Biobrane™ has been shown to reduce hospitalization time in the case of paediatric patients with second degree burn injuries.

Integra™ Dermal Regeneration Template is composed of a dermal layer made of porous bovine collagen and chondroitin-6-sulfate GAG, and an epidermal layer made of synthetic silicone polymer [25]. The silicone layer provides a functional barrier that is removed upon vascularization of the dermis, and replaced by a thin layer of autograft, while the dermal layer serves as a matrix for infiltration of fibroblasts and other cells from the wound bed. As the collagen-GAG matrix is populated by these cells, it is gradually degraded and replaced by newly synthesized collagen. Integra™ is widely used for the coverage of excised burn wounds, particularly in patients with large burns and limited autograft donor sites [25].

**Table 1:** List of commercially available skin substitutes.

Skin Substitute	Composition	Comments
Biobrane™	Outer epidermal analog—ultrathin silicone film; inner dermal analog—3D nylon filament with type I collagen peptides	Temporary wound dressing that is removed when wound is healed or when autograft skin is available
TransCyte™	Nylon mesh seeded with neonatal human foreskin fibroblasts that are destroyed before grafting	Temporary wound dressing upon which autografts are placed
Integra™	Dermal analog—bovine collagen and chondroitin-6-sulfate GAG; epidermal analog—silicone polymer	Silicone layer is removed upon vascularization of dermis, and replaced by a thin layer of autograft
Alloderm™	Human allograft skin that has been screened for transmissible pathogens, with all epidermal components and dermal cells removed	Grafted like dermal autograft and covered with a thin autograft
Dermagraft™	Bioabsorbable polygalactin mesh matrix seeded with human neonatal fibroblasts and cryopreserved	Matrix facilitates re-epithelialization by the patient’s own keratinocytes
Apligraf™	Bovine collagen gel seeded with neonatal foreskin fibroblasts and keratinocytes	Wound dressing with two different cell types
OrCel™	Type I collagen matrix seeded with neonatal foreskin fibroblasts and keratinocytes	Wound dressing with two different cell types
Epicel™	Sheets of autologous keratinocytes attached to petrolatum gauze support	Wound dressing with autologous cells
StrataGraft™	Full thickness skin substitute with dermal and fully differentiated epidermal layers	Made with naturally immortalized NIKS® keratinocyte cell line; contains two different cell types
Tiscover™ (A-Skin)	Autologous full thickness cultured skin for healing of chronic, therapy resistant wounds	Contains two different cell types
Permaderm™	Autologous tissue engineered skin consisting of epidermal and dermal cells	Contains two different cell types
denovoDerm™	Autologous dermal substitute	To be used in combination with split-thickness skin grafts
denovoSkin™	Autologous full thickness substitute consisting of dermal and epidermal layers	Contains two different cell types

Alloderm™, similar to Integra provides a matrix for dermal tissue remodelling [26]. It is composed of human allograft skin (cadaver skin) that has been screened for transmissible pathogens and processed to remove epidermal components and all dermal cells. The dermal cells are removed by detergent treatment followed by freeze drying, which preserves the matrix in a structural form similar to that of normal human dermis. A positive aspect of Alloderm™ is that it can be grafted like a dermal autograft and subsequently covered with a thin autograft. Also, since the allogeneic cells have been removed, it is not rejected by the immune system, which aids the regeneration of the underlying dermis. Alloderm™ has been successfully used in the resurfacing of full-thickness burn wounds in combination with an ultra-thin autograft which replaces the epidermis [1]. Preclinical studies have shown that Alloderm™ can be used for the repair of soft tissue defects such as in the case of abdominal wall reconstruction [27]. In addition to Alloderm™, other allogeneic skin substitutes are available as temporary wound covers, but they differ in matrix material composition and presence or absence of cells.

TransCyte™ is similar in composition to Biobrane™, and is used as a temporary cover for excised burns that await placement of autografts [28]. It consists of a nylon mesh seeded with fibroblasts cultured from neonatal human foreskin, which secrete extracellular matrix components and growth factors that aid the healing process. To reduce host immune response, before grafting, the fibroblasts are destroyed by a freezing process that preserves the tissue matrix and growth factors, and hence TransCyte™ has a possible benefit for wound healing over other strictly synthetic skin substitutes [28].

Dermagraft™ is prepared using human neonatal fibroblasts similar to TransCyte™, however the fibroblasts are cryopreserved to maintain cell viability and the matrix is made of bioabsorbable polygalactin mesh [23]. Dermagraft™ is used in the treatment of full-thickness foot ulcers, and functions by providing a dermal matrix that facilitates re-epithelialization by the patient’s own keratinocytes [23].

Apligraf™ is more advanced than TransCyte™ since it contains both fibroblasts and keratinocytes that are derived from neonatal foreskins [29]. A gel made of bovine collagen is used as the matrix for cell growth and differentiation. Apligraf™ has been useful in the treatment of venous leg ulcers and diabetic foot ulcers, by increasing the percentage of wounds healed and decreasing time required for wound closure [29]. Also, studies have reported the use of Apligraf™ for treatment of pediatric patients with various forms of epidermolysis bullosa, wherein no acute rejection reactions were observed but rather faster and less painful healing was noted compared to standard dressings [30,31].

OrCel™ is similar to Apligraf™ since it contains both fibroblasts and keratinocytes derived from neonatal foreskin, but uses a type I collagen sponge as the matrix [32]. It is used for grafting onto partial-thickness wounds, where it provides a favourable matrix for host cell migration. In a study that directly compared OrCel™ with Biobrane for the treatment of split-thickness donor site wounds, the OrCel™-treated sites had faster rates of healing and reduced scarring [32]. The improved healing was attributed to the presence of the collagen sponge, in combination with cytokines and growth factors produced by the viable allogeneic cells.

Epicel™, also known as cultured epidermal autografts, was the first commercially available autologous skin substitute. The ability to expand epidermal cells *in vitro* and produce autologous cultured epithelium was an important breakthrough in burn therapy [33], which led to the development of Epicel™. Epicel™ consists of sheets of autologous keratinocytes attached to a petrolatum gauze support, which is removed approximately 1 week after grafting [34]. It is used on patients with full-thickness burns covering greater than 30% TBSA and on patients with giant congenital nevus. Epicel™ is extremely valuable in patients with very large (>60% TBSA) burns where the donor site availability and quality is poor. In a study involving 30 extensively burned patients, Epicel™ was observed to provide permanent coverage of a mean TBSA of 26%, which represented a relatively high average take rate of approximately 69% of the area treated [34]. In another clinical study involving 28 patients with a mean TBSA of 52.2% and a mean total full thickness injury of 42.4% treated over a period of 5 years, Epicel™ had a mean take rate of 26.9% of the grafted area [35]. In these patients, overall mortality, hospitalization time and number of autograft harvests were not significantly different compared to a matched control population when Epicel™ was not available, suggesting that Epicel™ is likely more useful as a temporary wound dressing. Some of the other disadvantages associated with Epicel™ are its mechanical fragility, especially during the period of maturation of the dermal-epidermal junction, hyperkeratosis, contracture and scarring.

There are a few skin substitutes under development, some of them are discussed here. StrataGraft™ is a full thickness skin substitute consisting of a dermal component that contains human dermal fibroblasts and a fully-stratified epidermis derived from NIKS cells, a pathogen-free, genetically-stable human keratinocyte cell line [36]. StrataGraft™ has been granted an orphan product designation by the FDA for use in the treatment of partial and full-thickness burns. Tiscover™ is also a full thickness skin substitute, but is specifically being marketed for use in chronic therapy-resistant leg/foot ulcers. Permaderm™ is composed of autologous fibroblasts and keratinocytes cultured on a collagen substrate is that produces a skin substitute that contains both epidermal and dermal components. DenovoDerm™ and DenovoSkin™ are a dermal substitute and full-thickness skin substitute, respectively, and are currently undergoing trials.

Although a variety of skin substitutes are available for use depending on the requirement a systematic assessment of clinical and cost efficiency when used for burn treatment has not been reported. Recently, Hankin *et al.* evaluated clinical and cost efficacy of wound care matrices used for venous ulcers and found that the most expensive wound care matrices did not necessarily provide better clinical and cost efficacy [37]. 

## 7. Limitations of Commercially Available Skin Substitutes 

The commercially available skin substitutes have several limitations such as reduced vascularization, scarring, failure to integrate, poor mechanical integrity and immune rejection [24]. Skin substitutes when placed on wounds need to acquire blood supply rapidly for their long-term survival and integration into host tissue. Their inability to revascularize rapidly results in cell death and ultimate sloughing away from the host. Although some of the commercially available skin substitutes allow angiogenesis, the extent of vascularization is generally insufficient and needs to be further improved. Another important limitation is the development of scars at the graft margins after grafting, which results in a variety of functional, mechanical and aesthetic problems. Scar tissue is inferior in functional quality compared to native skin since it is less resistant to ultraviolet radiation, and does not grow back sweat glands and hair follicles unlike autografts. Furthermore, the costs associated with the use of the current skin substitutes is very high; for example, it is estimated that the cost for each 1% body surface area covered with Epicel™ is more than $13,000 [38].

## 8. Wound Healing and Fibrosis

Wound healing following burns or other injuries occurs by either regeneration or fibrosis [39]. Regeneration recapitulates the developmental processes that originally created the uninjured tissue, and reinstates the native tissue architecture, while fibrosis causes growth of connective tissue instead of the characteristic parenchymal tissue, resulting in the formation of dysfunctional and distorted tissue, commonly known as scar [39]. In humans, wound healing by regeneration is typical during prenatal development, but this ability is retained only to a limited extent during adulthood and therefore adult wounds often heal by fibrosis. Fibrotic conditions affect most organs and may cause either cosmetic and functional problems as seen in skin fibroproliferative disorders [hypertrophic scars and keloids] or organ failure as in idiopathic pulmonary fibrosis, liver cirrhosis, cardiovascular fibrosis, systemic sclerosis, and kidney fibrosis. Chronic fibrotic diseases are a leading cause of morbidity and mortality worldwide with current health statistical estimates of 45% of all deaths in the developed world [40]. Currently there are no clinically effective treatments for fibrotic diseases, thus there is an immediate need to develop innovative anti-fibrotic therapies. 

In normal skin, a fine balance between synthesis and degradation of collagen, the main component of the ECM, helps maintain physiological homeostasis. However during wound healing, the equilibrium is shifted towards accelerated collagen synthesis to aid tissue repair. In the case of fibrosis, collagen homeostasis is not restored at the culmination of the wound healing process, which results in excessive accumulation of collagen, patches of fibroblasts, hyper-cellularity, and a disorganized ECM. Fibrosis could lead to loss of proper function of the associated organ, and can be either local (hypertrophic scars and keloids) or systemic (systemic sclerosis) [40]. Hypertrophic scars, which are characterized by erythematous, raised, pruritic lesions of the healing skin, cause cosmetic and functional problems like color mismatch, stiffness, and rough texture, in addition to itching and pain [41]. Liver fibrosis interferes with drug metabolism causing accumulation of toxic metabolites, and lung fibrosis causes poor blood-gas exchange resulting in hypoxia [42]. In all, fibrosis has a significant impact on the outcome of wound healing and could impose increased health-care costs.

When the integrity of skin is compromised a cascade of events including formation of granulation tissue, re-epithelialization, and contraction of the underlying connective tissue is triggered, all of which ultimately lead to wound repair. The orchestrated tissue repair event is accompanied by recruitment of inflammatory cells to the wound site to fight possible infection. Fibrosis occurs as an aftermath of this inflammatory and connective tissue repair response to facilitate physiological repair of the body. It can be triggered by a variety of stimuli including persistent infections, autoimmune reactions, chemical insults, and tissue injury due to burns or other causes [40]. The key cellular mediator of fibrosis, myofibroblast, exhibits characteristics of both fibroblasts and smooth muscle cells. Myofibroblasts can be derived from resident mesenchymal cells, epithelial and endothelial cells, or fibrocytes [40]. They can be activated by a variety of mechanisms including Transforming Growth Factor (TGF)-β1, the most extensively studied pro-fibrotic cytokine or its downstream mediator, Connective Tissue Growth Factor (CTGF) and other autocrine factors, paracrine signals derived from lymphocytes and macrophages, and molecular patterns produced by pathogens [40]. They are highly contractile in nature, and generate connective tissue contracture and irreversible ECM remodelling producing stiff scars. Wound healing strategies aimed at modifying the local micro-environment from pro-fibrotic to anti-fibrotic may enable us to reduce myofibroblast-mediated fibrotic remodelling and achieve regenerative wound healing.

## 9. Tissue Engineering of Skin

Tissue engineering was originally described by Langer and Vacanti as an interdisciplinary field that applies the principles of engineering and life sciences toward the development of biological substitutes that restore, maintain, or improve tissue function [43]. Recently, it has been further defined as a field that understands and applies the principles of tissue growth to produce functional replacement tissue for clinical use [44]. Tissue engineering of skin was initially developed in the 1980s, with the primary motivation of providing coverage for extensive burn injuries in patients with insufficient sources of autologous skin for grafting. Tissue engineered skin products or skin substitutes can be cells delivered on their own, cells delivered within biomaterials, biomaterials used for replacement of dermis (with or without cells) or biomaterials used for replacement of both epidermis and dermis.

## 10. Tissue Engineered Skin

The commercially available skin substitutes do not provide for both the epidermal and dermal layers of the skin when placed on burn wounds. An exception to this is Epicel, a cultured epidermal autograft, which reconstructs the epidermis but does not provide for the dermis. Since the conventional skin substitutes do not aid the development of underlying connective tissues, the newly-formed skin lacks elasticity and mechanical stability [45]. Studies have shown that replacement of connective tissue aids healing of excised full thickness burns [46]. The fibrovascular connective tissue not only restores mechanical strength of the epidermis but also provides blood supply that nourishes it. Hence, repopulating the wound area with fibroblasts, endothelial and smooth muscle cells that form the connective tissue would facilitate formation of native-like skin. Tissue engineered skin is a promising alternative to conventional skin substitutes since it contains autologous fibroblasts and keratinocytes cultured on a scaffold. It is prepared using patient-derived fibroblasts and keratinocytes that are isolated from a split-thickness skin biopsy. Tissue engineered skin has been shown to effectively close full-thickness burn wounds since it provides both epidermal and dermal components that are required to achieve functional wound closure [23]. Further, clinical results have shown permanent replacement of both dermal and epidermal layers in a single grafting procedure [22,47,48,49]. Also, tissue engineered skin has been effective in treating burns of greater than 50% TBSA and giant congenital nevi in clinical studies [22,49,50].

## 11. Advantages of Tissue Engineered Skin

The main advantage of using tissue engineered skin for treatment of extensive burns is the decreased requirement of donor skin autografts, which is important because there are very limited skin graft donor sites in patients with extensive burns. There are reduced short-term complications of donor site wounds and long-term problems of development of scars and chronic wounds in patients. Use of tissue engineered skin has been reported to considerably decrease the length of hospitalization from the standard 1-1.5 hospital days per %TBSA full-thickness burn [51]. Tissue engineered skin has also been used as an adjunctive treatment for chronic wounds with allogeneic fibroblasts and keratinocytes from screened human cadaveric donors [48]. Another advantage is the presence of a large number of cells in tissue engineered skin, which facilitates rapid reformation of functional and protective barrier in the wound area by aiding regeneration of native-like skin.

## 12. Limitations of Tissue Engineered Skin

The development of tissue engineered skin is time consuming since extensive cell culture procedures are involved for the different cell types used. Cells for the epidermal and dermal components of tissue engineered skin usually require two to three weeks of cell culture before they are ready for grafting. This results in an increased turn over period for production of tissue engineered skin, which is a constraint for its regular use, and could be overcome, with technical advances in cell and tissue culture protocols. Although cultured skin substitutes work better than the conventional skin substitutes, they have some limitations. The currently available tissue engineered skin contain only two cell types, fibroblasts and keratinocytes, and hence lack the ability to form differentiated structures such as sweat and sebaceous glands, and hair follicles. In addition, melanocytes and Langerhans cells, adipose tissue and nerve supply are absent. Hence, these substitutes are unable to provide adequate temperature control, pigmentation, immune regulation, insulation and temperature and pressure sensation [23]. In order to overcome these anatomical limitations, which influence functional and cosmetic outcomes, and to increase homology to native skin, some studies have included additional cell types such as endothelial cells into tissue engineered skin [52,53]. However, these studies have not been successful due to technical difficulties such as slower growth of endothelial cells compared to fibroblasts, and higher rate of endothelial cell apoptosis. Further, melanocytes have been included in tissue engineered skin to overcome problems of irregular or absence of pigmentation due to insufficiency or lack of melanocytes. Preclinical studies where human melanocytes were selectively cultivated and added to tissue engineered skin showed uniform pigmentation although pigment intensity could not be regulated [54]. Recent studies have incorporated melanocytes, Langerhans cells and hair follicles into tissue engineered skin [55,56]. Hachiya *et al.* (2005) used mixed cell slurries containing keratinocytes and fibroblasts with melanocytes on the backs of severe immunodeficient mice, which gave rise to skin containing spontaneously sorted melanocytes [55]. Zheng *et al.* (2005) injected a mixture of neonatal dermal cells with epidermal aggregates into the dermis of nude mice and observed normal hair morphogenesis and hair follicle cycling within 8–12 days [56]. Recently, Böttcher-Haberzeth *et al.* (2013) showed that use of autologous melanocytes in bioengineered dermo-epidermal skin substitutes may potentially able to restore skin pigmentation in patients; in their study they co-cultured melanocytes, keratinocytes and fibroblasts on collagen gels, transplanted them onto full-thickness wounds on immunodeficient rats, and observed pigmentation irrespective of the keratinocyte/melanocyte ratio [57]. Further, they have very recently reported generation of lymphatic capillaries in collagen and fibrin hydrogels and were functional upon transplantation to immunocompromised rodents [58]. These studies show that it is possible to incorporate different cell types into tissue engineered skin in order to increase its homology to native skin and improve functional outcomes. Another important shortcoming of the currently used tissue engineered skin is that since it is made of heterogeneous dermal fibroblasts it is prone to pro-fibrotic remodelling and formation of HTS. Superficial dermal fibroblasts are anti-fibrotic and fewer in number compared to deep dermal fibroblasts that are pro-fibrotic and larger in number in the dermis. Heterogeneity among dermal fibroblasts will have serious implications on wound repair and healing following injury.

## 13. Tissue Engineered Skin—Potential for Promoting Anti-Fibrotic Healing

**Figure 2:**
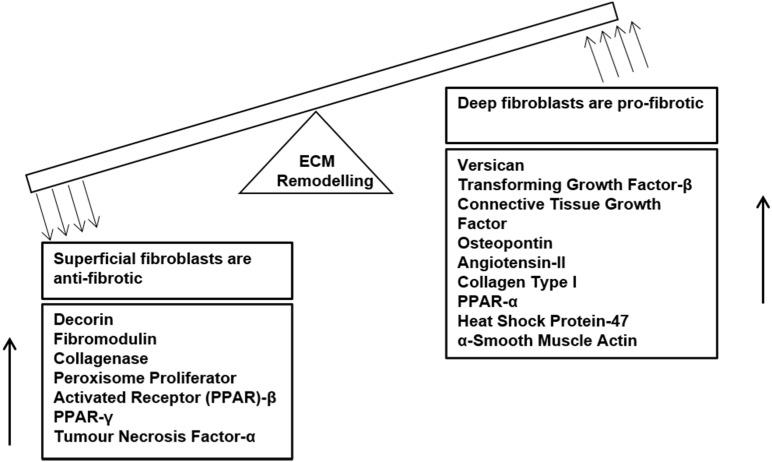
Dermal Fibroblast heterogeneity and their different roles in ECM remodelling. Superficial dermal fibroblasts negatively regulate fibrosis and have higher expression of anti-fibrotic genes, whereas deep dermal fibroblasts are promote fibrosis and have higher expression of pro-fibrotic genes.

As discussed previously, dermal fibroblasts are heterogeneous in nature; superficial and deep dermal fibroblasts exhibit physical and biochemical differences. Compared to superficial dermal fibroblasts, deep fibroblasts are larger, they proliferate slower in culture, produce more transforming growth factor (TGF)-β1 and its downstream mediator connective tissue growth factor (CTGF), collagen type I and osteopontin but less collagenase [59] (Figure 2). Deep fibroblasts also express less of the small proteoglycans, decorin and fibromodulin, but more of the large proteoglycan, versican, and differentiate more into myofibroblasts [60]. Superficial dermal fibroblasts were observed to be anti-fibrotic and behave similar to normal dermal fibroblasts, whereas deep fibroblasts were pro-fibrotic and behaved similar to hypertrophic scar fibroblasts [59]. Clinically, superficial wounds heal with minimum scarring, while deep wounds lead to formation of hypertrophic scars and contractures [61,62]. Further, we recently found that tissue engineered skin made of superficial fibroblasts and keratinocytes formed a continuous epidermis with increased epidermal barrier function and higher expression of epidermal proteins, keratin-5 and E-cadherin, compared to that with deep fibroblasts and keratinocytes, which had an intermittent epidermis [63]. In addition, tissue engineered skin with superficial fibroblasts and keratinocytes formed better basement membrane, and produced more laminin-5, nidogen, collagen type VII, compared to that with deep fibroblasts and keratinocytes. Overall, we found that tissue engineered skin with superficial fibroblasts and keratinocytes forms significantly better basement membrane with higher expression of dermo-epidermal adhesive and anchoring proteins, and superior epidermis with enhanced barrier function compared to that with deep fibroblasts and keratinocytes, or heterogeneous dermal fibroblasts and keratinocytes. Furthermore, co-culture with keratinocytes was found to reduce differentiation of deep fibroblasts to myofibroblasts in tissue-engineered skin constructs, but not in the case of superficial fibroblasts [64]. Previously, we had found that deep fibroblasts have higher cell surface expression of TGF-[beta] receptor II that has high ligand-binding affinity to TGF-β1 [65], which would be contributing to a fibrotic environment during wound healing. Another factor that is understood to contribute to the pro-fibrotic characteristics of deep dermal fibroblasts is their low Thy-1/CD90 activity [65]; Thy-1 deficient fibroblasts exhibit increased TGF-β1-mediated myofibroblast differentiation. Recently, CD26/DPP4 has been identified as a pro-fibrotic marker in murine skin [66]; inhibition of CD26 during wound healing was observed to reduce scarring. Further studies are required to determine if CD26 serves as a pro-fibrotic marker in the human population too.

## 14. Conclusions

When skin is compromised as a result of injury or trauma and the loss of skin is extensive, skin substitutes need to be used in order to re-establish the protective barrier of the skin. The most common mode of adult wound healing is by fibrosis, wherein connective tissue replaces the original parenchymal tissue and distorts the native architecture. Fibrosis affects most organs of the body, hypertrophic scar and keloid formation being the commonly observed fibrotic conditions affecting the skin. Of the skin substitutes for wound coverage, tissue engineered skin substitutes are preferred for tissue repair since they contain epidermal and dermal components embedded on artificial ECMs. Heterogeneity of dermal fibroblasts may have implications on post-burn wound healing, since superficial and deep dermal fibroblasts have been found to be anti-fibrotic and pro-fibrotic, respectively. The specific use of superficial fibroblasts in tissue engineered skin may thus be more beneficial to promote adhesion of newly-formed skin and to facilitate wound repair and regeneration in patients with extensive burn injuries due to its potential for anti-fibrotic healing. 
